# Effect of Season and Factory on Cheese-Making Efficiency in Parmigiano Reggiano Manufacture

**DOI:** 10.3390/foods8080315

**Published:** 2019-08-03

**Authors:** Piero Franceschi, Massimo Malacarne, Paolo Formaggioni, Claudio Cipolat-Gotet, Giorgia Stocco, Andrea Summer

**Affiliations:** Department of Veterinary Science, University of Parma, Via del Taglio 10, I-43126 Parma, Italy

**Keywords:** milk composition, Parmigiano Reggiano cheese, cheese-making efficiency, curd fines, cheese-making losses

## Abstract

The assessment of the efficiency of the cheese-making process (ECMP) is crucial for the profitability of cheese-factories. A simple way to estimate the ECMP is the measure of the estimated cheese-making losses (ECL), expressed by the ratio between the concentration of each constituent in the residual whey and in the processed milk. The aim of this research was to evaluate the influence of the season and cheese factory on the efficiency of the cheese-making process in Parmigiano Reggiano cheese manufacture. The study followed the production of 288 Parmigiano Reggiano cheese on 12 batches in three commercial cheese factories. For each batch, samples of the processed milk and whey were collected. Protein, casein, and fat ECL resulted in an average of 27.01%, 0.72%, and 16.93%, respectively. Both milk crude protein and casein contents were negatively correlated with protein ECL, r = −0.141 (*p* ≤ 0.05), and r = −0.223 (*p* ≤ 0.001), respectively. The same parameters resulted in a negative correlation with casein ECL (*p* ≤ 0.001) (r = −0.227 and −0.212, respectively). Moreover, fat ECL was correlated with worse milk coagulation properties and negatively correlated with casein content (r = −0.120; *p* ≤ 0.05). In conclusion, ECLs depend on both milk characteristics and season.

## 1. Introduction

The cheese-making process of rennet coagulated cheeses consists in the formation of a three-dimensional network of paracasein, in which fat globules and part of the milk whey are entrapped. The quantity of milk constituents recovered into cheese is strictly dependent on the quality of the milk (casein content, casein micelle structure, and integrity) and conditions of the cheese-making process (for example, pre-acidification of processed milk, type and quantity of rennet, cooking temperature, acidification of the cheese mass), and thus varies depending on the cheese type.

The assessment of the efficiency of the cheese-making process (ECMP) is crucial for the profitability of cheese-factories. The best way to quantify the ECMP is to measure the recovery of milk constituents into cheese through a mass balance determination. This can be obtained by measuring the quantity of a constituent in the processed milk and the resulting cheese. However, to perform this kind of analysis, it is necessary to also measure the weight of both the processed milk and cheeses. This is not always possible, especially in artisanal cheese factories, as those involved in Parmigiano Reggiano production, where the weight of the processed milk is estimated using a wooden measuring stick with a sensitivity, are not acceptable for research purposes.

Although less reliable than a mass balance, a rough and simple alternative way to estimate the ECMP is to measure the estimated cheese-making losses (ECL). In this method, the estimated loss of a milk constituent is expressed by the ratio between its concentration in the cheese whey (C-whey) (that remains in the vat after the extraction of the cheese mass) and that in the processed vat milk (V-milk). Consequently, the determination of ECL is easier and faster to perform, since for a single milk constituent it is necessary to only measure its concentration in V-milk and C-whey. No weight of milk, cheese, or whey is needed, and it is not necessary to sample and analyze the cheese. Moreover, to date, the concentration of most milk constituents can be rapidly assessed by applying the mid infrared technology (MIR) [1,2,3]. The MIR technology is widely used by a lot of laboratories which provide analysis and technological consulting services to dairy farms producing Parmigiano Reggiano cheese. In these laboratories, this kind of analysis is routine, cheap, and certified ISO/IDF. The ECLs have been used in several studies to estimate the effect of breed [4], storage conditions [5], and somatic cells [6,7] on efficiency in Parmigiano Reggiano cheese-making.

Parmigiano Reggiano is a hard, cooked, and long-ripened protected designation of origin (PDO) cheese made from raw milk, following a strict manufacture procedure [8]. In case of raw milk cheese, the quality of milk-in terms of chemical composition and microbial characteristics is one of the main factors influencing the efficiency of the cheese-making process. Seasonal variations of milk characteristics at the herd level were reported in several studies [9,10]. Significant variations of the chemical and microbial quality of milk employed for Parmigiano Reggiano cheese throughout the year have also been reported [11,12]. These variations could have repercussions on ECLs and curd fines, as reported by Formaggioni et al. [13] and by Franceschi et al. [5]. However, these papers considered a limited number of cheese-making trials and milk traits, since minerals were not taken into account and only two periods of the year were considered. Moreover, in the majority of PDO cheese manufacture, such as Parmigiano Reggiano, where milk standardization and automation of the processes are not implemented, a strong variability in the ECMP is expected among dairy farms [14]. However, the quantification of this variability has never been carried out.

The aim of this research was to evaluate the influence of the season and cheese-factory on ECLs on the quantity of curd particles lost in the whey (curd fines) in Parmigiano Reggiano cheese manufacture carried out in field conditions.

## 2. Materials and Methods

### 2.1. Cheese-Making Process

Cheeses were produced by the approved method of the Consortium [8,15]. A natural whey starter culture (about 2.5–3 L for every 100 kg V-milk), obtained by the spontaneous acidification of previous day milk whey (C-whey), was added to the V-milk before coagulation. It was then heated to 33 °C and clotted in 10–12 min with 2.5 g for every 100 kg V-milk of calf rennet (1:125,000 units). The curd was broken up into small granules (approximately the size of a rice grain) and cooked. During this operation, the temperature was increased in two steps to 55 °C within 10–15 min; during this phase the curd was stirred continuously. After cooking the broken-up curd particles, thy were deposited by simple decantation at the bottom of the vat, where they aggregated and blended together spontaneously. In this step, the temperature was 55–53 °C and the process lasted 45–60 min. The cheese mass was then removed from the vat, divided into two parts, and placed in special molds called “fascere” for two days. During this period, the cheese wheels were naturally cooled and periodically turned over to allow an homogeneous drying. Furthermore, pH decreased from about 6.0 (at the extraction from the vat) to about 5.1 at the end of the two days. This is related to the activity of thermophilic lactic bacteria added with the natural whey starter, which converts lactose to lactic acid. The cheese wheels were then placed into a saturated brine for a period of 20–25 days. Finally, the cheese entered the ripening phase, a process that lasts about 24 months, and, at the end of the ripening, the cheese wheels resulted in a cylindrical in shape, with a slightly convex side, 22–24 cm high, 40–45 cm diameter, weight 35–36 kg.

### 2.2. Experimental Design and Sampling Procedure

The research involved 288 Parmigiano Reggiano cheese-making trials, carried out in 3 cheese factories (CF1, CF2, and CF3), throughout two years.

Briefly, every cheese factory vat (that was filled with the milk of the same herd throughout the two years of the experimental period) was selected (6 vats in CF1, 3 in CF2, and 3 CF3). Each selected vat was sampled once a month, and all the vats of the same cheese factory were sampled on the same day. Each vat always contained the milk from the same farm. From each cheesemaking, samples of V-milk and C-whey were collected, following the International Dairy Federation standard [16]. V-milk samples were collected at the beginning of the cheese making process, before the addition of the natural whey starter culture. C-whey samples were collected after the extraction of the cheese mass and stirring of the whey.

### 2.3. Analytical Methods

The following traits were determined or calculated on both V-milk and C-whey. Total N (TN) and non casein N (NCN) were measured on milk and acid whey at pH4.6, respectively, by Kjeldahl, from which the values of crude protein (TN × 6.38/1000) and casein ((TN-NCN) × 6.38/1000) were calculated [7]. Fat content was assessed by the mid infrared method using a FT 6000 (Foss Electric, DK-3400 Hillerød Denmark); dry matter was measured after oven drying at 102 °C and ash was measured after muffle calcination at 530 °C [17]; total Ca and Mg were determined on a chloridric ash solution by atomic absorption spectrometry (AAS) with a wavelength reading at 422.7 and 285.2 nm, respectively; and total P was assessed on a chloridric ash solution following the colorimetric method [18] with a wavelength reading at 750.0 nm. Titratable acidity was measured only on V-milk by titration of 50 mL of milk with 0.25 N sodium hydroxide with the Soxhlet–Henkel method [7]. Rennet coagulation properties were also measured on V-milk, using Formagraph (Foss Electric, DK-3400 Hillerød Denmark) [4]. The analysis was performed adding 0.2 mL (1:100) of rennet solution (1:19,000; Chr. Hansen, I-20094 Corsico MI, Italy) to milk samples (10 mL). The rennet coagulation properties, milk clotting time, curd firming time, and curd firmness, were measured at 35 °C. Milk clotting time is the time from the addition of rennet to the onset of gelation. Curd firming time is the time from the onset of gelation till the signal attains a width of 20 mm. Curd firmness is the width of the signal 30 min after the addition of rennet. To record curd firming time values in milk samples that do not reach a width of 20 mm within 30 min, the analysis was prolonged to 60 min. The curd fines were determined in C-whey by the gravimetric method proposed by van den Berg et al. [19]. In this method, 250 of C-whey were centrifuged at 2000 g for 30 min. The pellet was resuspended in distilled water and filtered on a Whatman 40 filter paper. The filter was dried at 102 °C for 2 h and weighed.

ECLs of dry matter, protein, casein, fat, calcium, phosphorus, and magnesium were calculated as follows:

ECL = [C-whey] × 100/[V-milk]

where ECL is expressed as percentage; C-whey = concentration in whey, expressed as g/100 g (mg/100 g for Ca, P, Mg); V-milk = concentration in milk, expressed as g/100 g (mg/100 g for Ca, P, Mg).

### 2.4. Statistical Analysis

The significance of the differences between seasons and cheese-factories were tested by analysis of variance, using the software for statistical analysis SPSS (IBM SPSS Statics 23, Armonk, New York 10504-1722, NY, USA), according to the following univariate model:

Y_ijk_ = µ + S_i_ + C_j_ + ε_ijk_

where Y_ijk_ = dependent variable; µ = overall mean; S_i_ = effect of season (i = 1, …, 4; winter, from January to March; spring, from April to June; summer, from July to September, Autumn, from October to December); C_j_ = effect of cheese-factory (j = 1, …, 3; CF1, CF2, CF3); ε_ijk_ = residual error. The Bonferroni post-hoc test was employed to evaluate the significance of the differences between means. 

Data were also processed by the Pearson product moment correlation coefficient to measure the degree of linear relationship between milk constituents and ECLs.

## 3. Results

### 3.1. Overall Average Values and Descriptive Statistics

The descriptive statistics of V-milk characteristics, ECLs values, and curd fines content in whey are reported in Table 1. The Pearson product moment coefficient of correlations between the milk characteristics, ECLs values, and curd fines content are reported in Table 2.

The average contents of crude protein, casein, and fat in V-milk results were consistent with those reported by Formaggioni et al. [14] in a research carried out on 89 vat milk samples. Both contents results of the crude protein and casein in V-milk were negatively correlated with protein ECL and casein ECL. Moreover, casein content negatively correlated with fat ECL. This is in agreement with Malacarne et al. [4], who observed how milk with high casein content gives rise to a rennet curd with an improved capacity to entrap fat globules in the cheese matrix during coagulation. The casein ECL is lower if compared to those reported by Franceschi et al. [5], who found a casein ECL value of 1.25% for V-milk that was stored at 20 °C before processing. However, it is worth noting that Franceschi et al. [5] analysed only three samples collected in the winter season and three samples collected in the summer season. Protein ECL results were higher with respect to casein ECL, but showed a lower variability. The protein ECL average value was consistent with those reported by Franceschi et al. [5] (27.81%) and Summer et al. [7] (27.33%). The difference between the average values of protein ECL and casein ECL is due to milk whey proteins, which remain in the C-whey. Fat ECL showed a higher variability with respect to protein ECL and casein ECL. In this case, the average value found was consistent with the data reported by Franceschi et al. [5] (14.75%) and Summer, et al. [7] (14.95%). Fat ECL results correlated with the rennet coagulation parameters of V-milk. In particular, positive correlations were found with clotting time and curd firming time, while a negative correlation was evidenced with curd firmness. In fact, faster coagulating milk and firming curd give rise to higher curd firmness and, consequently, have an improved capacity to entrap fat globules into the paracasein matrix.

The curd fines are cheese particles that are too small to precipitate on the vat bottom, and therefore remain in suspension in the C-whey [20]. Consequently, they are not included in the cheese wheels. The curd fines content results were approximately twice than that reported by Franceschi et al. [5] (66.40 mg/kg), but this difference could be expected because, in their investigation, these Authors considered only six samples and the quantity of curd fines is generally small and its variability very high. The curd fines quantity in the C-whey results negatively correlated with the contents of crude protein and casein in V-milk and with the curd firmness, and positively correlated with the curd firming time. This is due to the fact that the higher the casein content is in the milk, the higher the crude protein content is [21] and the lower the results for the curd firming time [22], with a consequent higher curd firmness [21,22].

### 3.2. Seasonal Variations of ECLs

Seasonal variations of ECLs are shown in Table 3. The ECLs of dry matter, casein, fat, and calcium and the content of curd fines in the C-whey showed statistically significant differences among the seasons.

The estimated loss of dry matter result were lower in summer and higher in winter. It is worth noting that, although statistically significant, the differences were very small, amounting to approximately 1.5 percentage units. Additionally, casein ECL showed a very small variation, and result were higher in spring and lower in autumn. This is mainly due to the lactation stage of cows, which affects milk casein content with repercussions on casein ECL values. In fact, as reported by Summer et al. [11], during the spring season, most of cattle are in the early stage of lactation, which is characterized by a progressive increase of milk production and a decrease in milk protein content. In this season, milk samples showed the lowest average value of casein (2.40 g/100 g, data not shown in table). On the contrary, during the autumn season, most of cattle are in late lactation, which is characterised by a progressive decrease of milk production and an increase of milk protein and casein contents [11]. In this season, milk samples showed the highest casein content (2.52 g/100 g, data not shown in table).

Fat ECL results were lower during winter and spring and higher in summer and autumn. The high value of fat ECL during the summer season is due to the general worsening of the milk characteristics in this season, as reported by Summer et al. [11] and Bertocchi et al. [12]. The production area of Parmigiano Reggiano cheese during the summer period is characterised by a high temperature-humidity index [12]. This could induce heat stress conditions for the cow with an increase of the milk somatic cell content [11,12]. The increase of somatic cell content leads to a decrease of milk casein content [7], titratable acidity value [7,11], and alteration of milk mineral content and salts distribution [7,23], with a worsening of rennet coagulation properties [24,25] and an increase of fat losses [7,13].

Curd fines results were lowest in winter and highest in spring. This is in contrast with reports from Summer et al. [20], who did not find significant differences among seasons. However, Summer et al. [20] collected the samples from May to January and considered only two seasonal categories, namely Spring–Summer and Autumn–Winter. The seasonal trend of curd fines, although significant, is difficult to explain because the quantity of curd fines that remain in the C-whey is affected by many factors, such as milk casein content, curd firming time, and curd firmness [5,13,20]. For example, in a research carried out on 102 milk samples, Formaggioni et al. [13] showed a significant correlation between curd fines content of C-whey, curd firming time, and curd firmness of milk. In the present research, milk casein content and rennet coagulation properties (data not shown) showed different seasonal trends and, it is likely that, since all these parameters influenced the curd fines content in different measures, curd fines did not show a clear seasonal trend.

### 3.3. Difference of ECLs among Cheese Factories

Difference of ECL values among cheese factories are shown in Table 4. The cheese-making losses of dry matter, fat, protein, casein, phosphorus, ash, and curd fines showed significant differences between the cheese factories. Compared to the other two cheese factories, CF3 showed higher values of protein, casein, fat, and ash ECLs. This in turn affected the dry matter ECL that was significantly higher in this cheese-factory. It is not easy to explain the difference of the ECLs based on CF3 milk characteristics, as they were not always the worst among cheese factories. Even when considering the level of cheese production and the location of the three cheese factories, differences in ECLs are hard to explain. In fact, CF1 is characterized by a higher production and is located in the plain zone; CF2 is characterised by a small production and is located in the hill zone; and the CF3 is characterised by a small production, similar to the CF2, and is located in the plain zone, like the CF1. The difference in ECLs among cheese factories can be explained with differences in the technological process. Parmigiano Reggiano cheese is still a craftsmanship product, not a standardized product, and only the crucial technological steps are reported in the Parmigiano Reggiano cheese disciplinary. A lot of variability can be observed between different cheese factories on the duration of technological steps, the dimensions of curd granules during the curd broken step, and the quantity of whey starter cultures that are added to the V-milk in the pre-acidification milk step. 

## 4. Conclusions

In conclusion, the two most relevant parameters of the cheese-making losses are protein and fat ECLs; they strictly depend on milk characteristics, in particular, milk fat, casein contents, and rennet coagulation properties. Since the season affects the milk composition and the rennet coagulation properties, it also influences the ECL. Finally, the differences in the technology of milk transformation in cheese that exists among the cheese factories strongly affected the ECLs. The estimated cheese-making losses of protein and fat can be used as an instrument for the control of the technological process.

## Figures and Tables

**Table 1:** Descriptive statistics of vat milk characteristics and estimated cheese-making loss (ECL) values from 288 Parmigiano Reggiano cheese-making trials.

		Mean		SD ^1^	Minimum	Maximum	CV ^2^ (%)
Vat milk characteristics							
Dry matter	g/100 g	11.73	±	0.32	10.67	12.45	2.70
Crude protein	g/100 g	3.18	±	0.12	2.76	3.47	3.77
Casein	g/100 g	2.46	±	0.10	2.11	2.66	3.94
Fat	g/100 g	2.68	±	0.21	2.02	3.13	7.86
Fat to casein ratio	Value	1.09	±	0.08	0.85	1.27	7.04
Ash	g/100 g	0.73	±	0.01	0.69	0.78	2.02
Calcium	mg/100 g	119.59	±	5.31	109.22	138.46	4.44
Phosphorus	mg/100 g	88.62	±	3.29	77.90	97.90	3.71
Magnesium	mg/100 g	10.67	±	0.76	9.23	15.14	7.17
Titratable acidity	°SH/50 mL	3.29	±	0,11	2.95	3.60	3.37
Clotting time	min	18.52	±	2.20	11.50	24.00	11.89
Curd firming time	min	7.01	±	2.80	2.75	11.25	39.94
Curd firmness	mm	26.25	±	6.10	9.44	43.48	23.23
ECLs ^3^							
Dry matter	%	66.91	±	3.12	58.77	75.18	4.66
Protein	%	27.01	±	0.93	22.44	31.59	3.45
Casein	%	0.72	±	0.05	0.10	3.50	6.94
Fat	%	16.93	±	3.59	10.31	27.78	21.21
Ash	%	75.42	±	1.57	70.07	84.57	2.08
Calcium	%	36.51	±	2.73	28.07	44.08	7.48
Phosphorus	%	50.87	±	2.25	44.43	58.76	4.42
Magnesium	%	76.54	±	4.59	54.83	88.57	6.00
Curd fines	mg/kg	122.01	±	66.63	9.30	428.00	54.61

^1^ Standard deviation; ^2^ Coefficient of variation; ^3^ Estimated cheese-making losses, expressed as the % of ratio between the concentrations in the residual cheese whey and vat milk.

**Table 2:** Pearson product moment correlation coefficient (r) between the milk characteristics and the estimated cheese-making loss (ECL) values and curd fines. Only significant correlations (*p* < 0.05) are reported.

	ECLs ^1^ (%)							
	Protein		Casein		Fat		Curd Fines	
	r	*p* ^2^	r	*p* ^2^	r	*p* ^2^	r	*p* ^2^
Dry matter			−0.112	*			−0.114	*
Crude protein	−0.141	*	−0.227	***			−0.185	**
Casein	−0.223	***	−0.212	***	−0.120	*	−0.195	***
Fat								
Clotting time					0.141	*		
Curd firming time					0.169	**	0.109	*
Curd firmness					−0.176	**	−0.152	*

^1^ Cheese-making loss for a milk constituent is expressed as the % ratio between the concentrations in the residual cheese whey and vat milk; ^2^ *p*-value: * *p* ≤ 0.05; ** *p* ≤ 0.01; *** *p* ≤ 0.001.

**Table 3:** Seasonal variation of estimated cheese-making loss values and curd fines (least square means values).

		Winter *n* ^1^ = 72		Spring *n* ^1^ = 72		Summer *n* ^1^ = 72		Autumn *n* ^1^ = 72		SE ^2^	*p* ^3^
Dry matter	%	67.61	b	66.94	a,b	66.08	a	67.01	a,b	0.35	*
Crude protein	%	27.05		26.82		26.95		27.21		0.11	NS
Casein	%	0.79	b,c	0.87	c	0.63	a,b	0.58	a	0.06	**
Fat	%	15.94	a	16.51	a,b	17.52	b,c	17.74	c	0.35	**
Ash	%	75.43		75.76		75.42		75.06		0.19	NS
Phosphorus	%	50.84		51.18		50.91		50.54		0.28	NS
Calcium	%	36.96	b	37.13	b	36.83	b	35.00	a	0.42	***
Magnesium	%	76.70		76.69		76.43		76.41		0.83	NS
Curd fines	mg/kg	104.17	a	137.27	b	124.89	a,b	121.68	a,b	5.81	*

^1^ Number of samples; ^2^ Standard error of the mean; ^3^ *p*-value: a, b, and c are different for *p* ≤ 0.05; NS, *p* > 0.05; * *p* ≤ 0.05; ** *p* ≤ 0.01; *** *p* ≤ 0.001.

**Table 4:** Differences among cheese factories in estimated cheese-making loss values and curd fines.

		Cheese Factory 1 *n* ^1^ = 144			Cheese Factory 2 *n* ^1^ = 72			Cheese Factory 3 *n* ^1^ = 72			*p* ^4^
		LSMean ^2^	SE ^3^		LSMean ^2^	SE ^3^		LSMean ^2^	SE ^3^		
Dry matter	%	67.68	0.25	b	65.84	0.36	a	66.40	0.36	b	***
Crude protein	%	26.97	0.07	a	26.89	0.10	a	27.25	0.10	b	*
Casein	%	0.63	0.04	a	0.75	0.06	a,b	0.85	0.06	b	*
Fat	%	15.53	0.25	a	16.71	0.36	b	19.99	0.36	c	***
Ash	%	75.75	0.13	b	74.83	0.18	a	75.33	0.18	a,b	***
Phosphorus	%	51.24	0.19	b	50.53	0.27	a	50.45	0.27	a	*
Calcium	%	36.90	0.26		35.94	0.36		36.19	0.36		NS
Magnesium	%	77.21	0.46		75.74	0.64		76.09	0.64		NS
Curd fines	mg/kg	111.06	5.42	a	127.21	7.78	a,b	139.07	7.78	b	*

^1^ Number of samples; ^2^ Least square means values; ^3^ Standard error of the mean; ^4^ *p*-value: a, b, and c are different for *p* ≤ 0.05; NS, *p* > 0.05; * *p* ≤ 0.05; *** *p* ≤ 0.001.
